# Supplementary material for: cAMP signaling regulates DNA hydroxymethylation by augmenting the intracellular labile ferrous iron pool
Source: eLife. 2017 Dec 14;6:e29750. doi: 10.7554/eLife.29750 (PMC5745079; doi:10.7554/eLife.29750)
Supplement: Figure 1—source data 1. [file elife-29750-fig1-data1.docx]

**Figure 1** **− *Source data 1*.** Primers used for quantitative RT-PCR.

| **Gene** | **Forward (5′→3′)** | **Reverse (5′→3′)** |
| --- | --- | --- |
| *Tet1* | CATGGAGACTAGGTATGGCCAGAAGG | CTTCTTCTAATCACCCACTTGGCGAC |
| *Tet2* | GAACAATCATGGAAGAAAGGTTTGGAGAG | GTACTTGACCTCCGATACACCCATTTAG |
| *Tet3* | GGGAACTCATGGAGGATCGGTATGGAG | GTGTGTGTCTTCGGATCACCCACTTG |
| *Rapgef2* | TAGAGGGTGACTTGCCATCG | TCTGCAGGAAGTGAGTGTTTC |
| *Sdha* | GAAGGCATCCGCTAAAGTTTCAGACGCG | GCAGCATTGATACCTCCCTGTGCTGC |

**Note:** *Sdha* was used as a loading control.
