# Supplementary material for: cAMP signaling regulates DNA hydroxymethylation by augmenting the intracellular labile ferrous iron pool
Source: eLife. 2017 Dec 14;6:e29750. doi: 10.7554/eLife.29750 (PMC5745079; doi:10.7554/eLife.29750)
Supplement: Figure 5—source data 1. [file elife-29750-fig5-data1.docx]

**Figure 5 − *Source data 1*.** Fragments per kilobase per million (FPKM) of CNG and Rapgef genes in Schwann cells.

| Gene Name | Control-1 | Control-2 | Control-3 | Control Average | cAMP-1 | cAMP-2 | cAMP-3 | cAMP Average |
| --- | --- | --- | --- | --- | --- | --- | --- | --- |
| Cnga1 | 0.00 | 0.02 | 0.00 | 0.01 | 0.02 | 0.00 | 0.00 | 0.01 |
| Cnga2 | 0.00 | 0.00 | 0.00 | 0.00 | 0.00 | 0.00 | 0.00 | 0.00 |
| Cnga2 | 0.00 | 0.00 | 0.00 | 0.00 | 0.00 | 0.00 | 0.00 | 0.00 |
| Cnga3 | 0.00 | 0.00 | 0.00 | 0.00 | 0.00 | 0.00 | 0.00 | 0.00 |
| Cnga4 | 0.14 | 0.10 | 0.09 | 0.11 | 0.01 | 0.02 | 0.00 | 0.01 |
| Cngb1 | 0.24 | 0.23 | 0.11 | 0.19 | 0.17 | 0.24 | 0.21 | 0.20 |
| Cngb3 | 0.00 | 0.00 | 0.00 | 0.00 | 0.00 | 0.00 | 0.00 | 0.00 |
| Rapgef1 | 8.86 | 9.77 | 7.18 | 8.60 | 5.26 | 5.13 | 5.31 | 5.23 |
| Rapgef2 | 27.82 | 28.13 | 26.69 | 27.55 | 21.33 | 18.11 | 20.35 | 19.93 |
| Rapgef3 | 0.00 | 0.00 | 0.00 | 0.00 | 0.02 | 0.01 | 0.03 | 0.02 |
| Rapgef4 | 0.14 | 0.04 | 0.09 | 0.09 | 0.08 | 0.05 | 0.05 | 0.06 |
| Rapgef5 | 0.01 | 0.01 | 0.04 | 0.02 | 0.01 | 0.00 | 0.00 | 0.00 |
| Rapgef6 | 8.01 | 9.00 | 7.61 | 8.21 | 11.28 | 11.49 | 11.16 | 11.31 |

**Note:** The RNA-seq data shows that the expression of CNG genes, Rapgef3 and Rapgef4 are extremely low in both control and cAMP-treated Schwann cells.
