# Supplementary material for: cAMP signaling regulates DNA hydroxymethylation by augmenting the intracellular labile ferrous iron pool
Source: eLife. 2017 Dec 14;6:e29750. doi: 10.7554/eLife.29750 (PMC5745079; doi:10.7554/eLife.29750)
Supplement: Figure 8—source data 1. [file elife-29750-fig8-data1.docx]

**Figure 8 − *table supplement 1*.** Impact of cAMP treatment on both transcription and 5hmC levels of myelin-related genes in Schwann cells.

| **Gene Name** | **Indicative Schwann**  **Cell phenotype** | **Control**  **(FPKM)** | **cAMP**  **(FPKM)** | **Fold Change** | ***P*-value** | **5hmC change** | |
| --- | --- | --- | --- | --- | --- | --- | --- |
|  |  |  |  |  |  | **Promoter** | **Gene Body** |
| Cdh1 | Myelinating | 0.03 | 32.71 | 1193.5 | <1E-300 | No Change | Up |
| Mag | Myelinating | 0.33 | 99.16 | 302.1 | <1E-300 | Up | No Change |
| Prx | Myelinating | 2.48 | 639.87 | 257.6 | <1E-300 | Up | Up |
| Fa2h | Myelinating | 0.31 | 12.00 | 39.2 | 5.11E-168 | Down | Up |
| Mbp | Myelinating | 103.14 | 2079.57 | 20.1 | <1E-300 | Up | Up |
| Egr2 | Myelinating | 4.58 | 77.10 | 16.8 | 7.32E-149 | No Change | Up |
| Pmp22 | Myelinating | 220.81 | 1541.04 | 6.9 | 1.01E-157 | No Change | Up |
| Ugt8 | Myelinating | 56.73 | 209.02 | 3.7 | 4.48E-60 | No Change | Up |
| Pou3f2 | Myelinating | 3.50 | 12.28 | 3.5 | 5.80E-32 | No Change | No Change |
| Mpz | Myelinating | 1893.53 | 5532.61 | 2.9 | 4.42E-57 | No Change | No Change |
| Plp1 | Myelinating | 286.05 | 546.21 | 1.9 | 2.58E-20 | No Change | No Change |
| Cnp | Myelinating | 803.29 | 1076.55 | 1.3 | 1.49E-09 | No Change | No Change |
| Erbb3 | Myelinating | 221.59 | 290.59 | 1.3 | 1.39E-06 | Up | Up |
| Cdh19 | Precursor | 386.59 | 156.66 | 0.4 | 9.11E-22 | No Change | Up |
| Nes | Immature | 163.55 | 60.19 | 0.3 | 2.55E-23 | No Change | No Change |
| Jun | Immature | 395.06 | 54.11 | 0.1 | 5.36E-126 | No Change | No Change |
| Gfap | Nonmyelinating | 47.83 | 4.83 | 0.1 | 2.87E-101 | No Change | No Change |
| Ngfr | Immature | 696.47 | 43.00 | 0.06 | 9.00E-257 | No Change | Up |
| L1cam | Immature | 131.80 | 5.67 | 0.04 | 1.19E-268 | No Change | No Change |

**Note:** FPKM: Fragments per kilobase per million. Cdh1: Cadherin-1 (E-Cadherin), Mag: Myelin associated glycoprotein, Prx: Periaxin, Fa2H: Fatty acid 2-hydroxylase, Mbp: Myelin basic protein, Egr2: Early growth response 2 (Krox20), Pmp22: Peripheral myelin protein 22, Ugt8: UDP glycosyltransferase 8, Pou3f2: POU domain class 3 transcription factor 2 (Brn2), Mpz: Myelin protein zero, Plp1: Proteolipid protein 1, Cnp: 2',3'-cyclic nucleotide 3' phosphodiesterase, ErbB3: erb-b2 receptor tyrosine kinase 3, Cdh19: Cadherin-19, Nes: Nestin, Jun: Jun proto-oncogene AP-1 transcription factor subunit (c-Jun), GFAP: Glial fibrillary acidic protein, Ngfr: Nerve growth factor receptor, L1cam: L1 cell adhesion molecule
